# Probiotic Characteristics of *Lactiplantibacillus plantarum* CECT 9435 and Its Survival and Competitive Properties Under Simulated Conditions of the Child Gut Microbiota

**DOI:** 10.1007/s12602-024-10280-w

**Published:** 2024-05-03

**Authors:** Teresa Requena, M. Carmen Martínez-Cuesta, Rosa Aznar, M. Luz Mohedano, Paloma López, Patricia Ruas-Madiedo

**Affiliations:** 1 Department of Food Biotechnology and Microbiology, BFBL Group, Instituto de Investigación en Ciencias de la Alimentación (CIAL), CSIC, Madrid, Spain; 2 Department of Microbiology and Ecology and Spanish Collection of Type Cultures (CECT), University of Valencia, Valencia, Spain; 3 Department of Microorganisms and Plant Biotechnology, Margarita Salas Center for Biological Research (CIB), CSIC, Madrid, Spain; 4 MicroHealth Group, Instituto de Productos Lácteos de Asturias (IPLA), CSIC, Villaviciosa, Asturias, Spain

**Keywords:** Probiotic, *Lactiplantibacillus plantarum*, Fortified food, Child gut microbiota, HT29

## Abstract

Probiotics are valuable microorganisms effective in reducing malnutrition-related infections in children. In this work, a collection of lactobacilli strains representative of traditional Andean fermented beverages was in vitro screened for their capability to survive the gastrointestinal transit, to adhere to the intestinal epithelium and to compete under simulated conditions of the child gut microbiota. The results allowed the selection of the riboflavin overproducing strain *Lactiplantibacillus plantarum* CECT 9435 based on its good rate of survival under in vitro gastrointestinal conditions when included in a food matrix representing the fortified food supplement Incaparina. The strain also showed good adhesion to HT29 cells producing mucus and outstanding performance in *E. coli* competition for the adhesion to this epithelial cell line. *L. plantarum* CECT 9435 gut performance was also evaluated in the child intestinal microbiota simulated in a dynamic gut model (BFBL simulator). The viability of the probiotic candidate in the gut conditions was high during the 7-day intervention period, reaching over 1 × 10^7^ counts in each of the reactors simulating the three colonic regions. The transient viability of *L. plantarum* CECT 9435 within the child gut microbiota and its adhesion capacity to intestinal cells could facilitate the strain potential benefits as probiotic added to fortified supplementary foods destined to malnourished children.

**Supplementary Information:**

The online version contains supplementary material available at 10.1007/s12602-024-10280-w.

## Introduction

Acute infectious diarrhoea and undernutrition are leading killers of children under five years of age in low- and middle-income countries, despite the availability of treatment solutions [1]. Both conditions are interrelated, since undernutrition predisposes children to a greater incidence and duration of diarrhoea, and are associated with the absence of the beneficial mature anaerobic gut microbiota [2]. Some probiotic interventions carried out with well-nourished children in high-income societies, where infectious gastroenteritis is typically mild and self-limiting, have shown that the use of probiotics may not be critical [3, 4]. However, studies performed in hospitals from low and middle-income countries have reported that probiotics, mainly lactobacilli and bifidobacteria strains, seem to reduce all-cause mortality in babies born with low birth weight and/or preterm [5]. Moreover, the administration of probiotics to prevent the colonization by opportunistic or pathogenic microorganisms has demonstrated to be effective particularly in undernourished children [6].

The potential of probiotics to reduce acute-infectious diarrhoea can be mediated by direct competition against pathogens and indirectly via interaction with commensal gut microbiota or mediated by immune system modulation [7]. Effective ways of competition can be as follows: (i) the improvement of the barrier effects of the mucus, (ii) production of antimicrobial compounds, (iii) competition for binding sites in mucin or epithelial cells and (iv) inhibition of the adhesion of intestinal pathogens [8, 9]*.* The survival and colonization of ingested probiotics inside the gut microbiota environment generally experience the resistance to colonization by commensal bacteria, which originates transiently faecal recovery of the strains during supplementation and their subsequent cleansing [10, 11]. Nevertheless, the impact of probiotics does not necessarily reside in their ability to graft in the microbiota, but rather in sharing genes and metabolites able to interact with the host [12]*.* Moreover, during transit, probiotics can induce a transitory, individualized impact on mucosal community structure and gut transcriptome [13].

The characterization of natural fermentative strains portraying particular traits can guarantee the heritage conservation of well-adapted probiotics able to develop high nutritional value, vegetable-based fermented foods [14]. The lactic acid bacteria most frequently isolated from traditional fermented vegetables are *Lactiplantibacillus plantarum* strains [14, 15]. Some of them might possess several antimicrobial characteristics, which are mainly exerted by bacteriocins, followed by organic acids or acidic conditions, and biosurfactants such as glycoproteins [16]. The present study was carried out under the framework of the ProInfant-CYTED project, a multidisciplinary and collaborative work of several Latin American, Spanish, and Italian research groups, aimed to develop high nutritional value, vegetable-based fermented foods, containing well-adapted probiotic strains able to reduce malnutrition-related diseases, such as diarrhoea and respiratory infections [17]. In the framework of this multidisciplinary project, amongst other results, lactic acid bacteria adapted for cereal-based food fermentation have been characterized [14] and an intervention trial with the probiotic selected in this study was performed in Guatemala with chronical malnourished children (results not published). For the probiotic selection, a collection of eight strains obtained in the framework of a previous research project (MICROANDES “Microbiota of Andean Food: tradition for healthy products” ref. 247,650 FP7-PEOPLE-2009-IRSES) were in vitro screened for their capability to survive the gastrointestinal transit and to adhere to the intestinal epithelium, also inhibiting the adhesion of *Escherichia coli*. Finally, in order to reproduce the interaction with the children gut microbiota, a selected *L. plantarum* strain was supplied to the dynamic colonic simulator BFBL gut model, together with residual content of “Incaparina® fortificada, con leche”, a commercial maize and soya-based food matrix, containing milk and fortified with vitamins, iron and zinc, that was initially developed by the Institute for Nutrition of Central America and Panama (INCAP, Guatemala).

## Materials and Methods

### Strains and Culture Media

The lactobacilli strains used in this study are listed in Table 1, which were selected from a wide collection of isolates from Andean traditional fermented foods [18]. In addition, two probiotic strains, *Lactiplantibacillus plantarum* 299 V (DSM9843) and *Lacticaseibacillus rhamnosus* GG (LMG18243), acquired from culture collections, were used as reference. Stocks stored at − 80 °C were spread on the surface of agar-MRS (Biokar Diagnostics) and cultured for at least 48 h at 32 °C. For the standardisation of cultures, a single colony was inoculated in MRS broth and incubated for 24 h. Finally, 2% of this culture was used to inoculate fresh MRS that was cultivated for 18 ± 1 h. These standardised cultures were used for different experiments, described next, towards the selection of a probiotic candidate to be studied in the BFBL dynamic gut model.

**Table 1:** Main characteristics of the strains used in this work from the species *Lactiplantibacillus plantarum* and *Lacticaseibacillus rhamnosus*

**Species**	**CECT code**	**Other codes /food origin**	**Characteristics**
*L. plantarum*	CECT 8962	M5MA1	Riboflavin producer; NCCFS* antibacterial activity
*L. plantarum*	CECT 9434	M5MA1-mut or M5MA1-B2 (from corn-Chicha)	Riboflavin overproducer; NCCFS* antibacterial activity
*L. plantarum*	CECT 8965	M9MG6	Riboflavin producer; NCCFS* protease-sensitive antibacterial activity
*L. plantarum*	CECT 9435	M9MG6-mut or M9MG6-B2 (from corn-Chicha)	Riboflavin overproducer; NCCFS* protease-sensitive antibacterial activity
*L. plantarum*	CECT 8963	M9MM1 (from corn-Chicha)	Riboflavin producer; NCCFS* protease-sensitive antibacterial activity
*L. plantarum*	CECT 9571	CECT 9492-B2 (roseoflavin-derived from CECT9492)	Riboflavin overproducer; high adhesion to Caco2; resistant to gastrointestinal conditions
*L. plantarum*	CECT 9491	CRL1905 or QY89 (from Quiona)	Riboflavin and folate producer; GABA producer
*L. rhamnosus*	CECT 9490	CRL1891 or A29 (from Amaranto)	Riboflavin and folate producer
*L. rhamnosus*	LMG 18243	GG	Reference probiotic
*L. plantarum*	DSM 9843	299 V	Reference probiotic

^*^NCCFS neutralized concentrated cell-free supernatants

### Selection of the Probiotic Candidate

#### Survival of Strains in a Food Carrier Submitted to Simulated Static Digestion

A food matrix, resembling the composition of the fortified Incaparina®-milk, was prepared according to the procedure described by Russo et al. [19] with minor modifications. In short, 250 mL of a water-beverage containing 37.5 g of powder (65% corn flour, 20% skimmed milk, and 15% soy flour) were well homogenized and pasteurized in a water bath for 10 min at 95 °C, to finally cooling down at 37 °C. Standardised lactobacilli cultures (100 mL) of the ten strains were obtained in MRS as previously described and washed twice with PBS solution. The harvested pellets were well resuspended in the same volume of the pasteurized food matrix. Counts of the viable bacteria in this food matrix (initial sample) were carried out by plating into agar-MRS serial dilutions, made on Ringer 1/4 (Merck), following incubation at 32 °C for 48 h.

Food-bacterial suspensions were submitted to the Infogest static in vitro simulation of gastrointestinal food digestion [20, 21] with some modifications [14]. The experiment consisted of four sequential steps (oral phase, gastric phase, duodenal phase, and intestinal phase). In each of them, a sample was taken to determine the viable bacteria by enumeration on agar-MRS as indicated above. The preparation of the oral, gastric and intestinal simulated fluids was performed according to the formulation of the Infogest protocol. In the first step, i.e. oral phase, 5 g of the food-bacterial suspensions was mixed with the same volume of the simulated salivary fluid (pH 7) containing α-amylase solution (75 U/mL final concentration) and maintained at 37 °C for 2 min in a rotatory incubator. Then, 5 mL of this oral food-bolus was mixed with the same volume of the simulated gastric fluid (pH 3.0) containing pepsin solution (2000 U/mL) and shacked for 2 h at 37 °C. For the duodenal step, 5 mL of this food-gastric chyme was mixed with the simulated intestinal fluid (pH 7.0) containing a pancreatin solution (100 U/mL trypsin activity) and 10 mM of bile salts. The mixture was placed in the shaking incubator for 10 min at 37 °C. Finally, in the intestinal phase, 5 mL of the duodenal-digested food was mixed with two volumes of the simulated intestinal fluid, in order to dilute the pancreatin and the bile salts (to around 3 mM), and the mixture was kept for 2 h under the same conditions. The pH of all mixtures in the different steps was checked and adjusted when necessary (see Supplementary Table S1). All reagents used in the study were from Sigma (Sigma-Merck), and the experiments were repeated at least in duplicate. The survival of each lactobacilli strain was calculated as the percentage of the CFU/mL obtained after each phase with respect to the CFU/mL of the initial bacterial suspension in the food matrix.

#### In Vitro Adhesion to the Intestinal Epithelium

The adhesion to the intestinal epithelium of the eight strains from Andean food origin, together with the two probiotics strains used as reference, was tested on the cell line HT29 (ECACC 91072201, European Collection of Cell Cultures, UK), according to procedures previously described [14]. Briefly, bacterial cultures (10 mL) were washed twice with PBS and resuspended in 1 mL (at 10^8^ CFU/mL) of the complete (without antibiotics) McCoy’s medium (Sigma) specific for this cell line. Then, 13 ± 1-day-old HT29 cell monolayers, seed on 12-well plates, were gently washed with Dulbecco PBS (Sigma) before adding the bacterial suspensions. Monolayers, with bacteria-HT29 cell ratio around 10:1, were incubated for 1 h at 37 °C/5% CO_2_. Supernatants were removed and gently washed twice with Dulbecco PBS before adding 0.5 mL of 0.25% EDTA-trypsin (Sigma). Plates were incubated under same conditions for 10–15 min, to favour the release of the cells from the monolayer, and then 0.5 mL of complete-McCoy’s medium was added. Bacterial counts of the initial cells added as well as those adhered to HT29 cells were enumerated by plating in agar-MRS. The percentage of adhesion was calculated as the CFU adhered bacteria divided by the CFU of added bacteria. This procedure was performed with triplicated cultures of each lactobacilli strain.

#### Competition with *E. coli* for the In Vitro Adhesion to the Intestinal Epithelium

The enterohemorrhagic *E. coli* CECT4267 (O157:H7) was routinely grown in LB broth at 37 °C under stirring. The capability of the lactobacilli to compete with *E. coli* for the adhesion to HT29 monolayers was evaluated as previously reported [22]. In short, lactobacilli and *E. coli* suspensions were separately prepared in McCoy’s medium at concentration about 1 × 10^8^ CFU/mL, and finally mixed at ratio 1:1. Afterwards, 1 mL of this mixture was added to differentiated (13 ± 1 days) HT29-cell monolayers seed on 12-well plates (bacteria-HT29 cell ratio around 10:1) which were incubated for 1 h at 37 °C/5% CO_2_. After this time, plates were treated as described in the previous section to breakdown the monolayer and *E. coli* counts in the initial bacterial suspension added and in the supernatants of trypsinized monolayers (*E. coli* adhered) ware performed on VRBA (Merck). The percentage of adhesion of the pathogen in the absence and in the presence of the lactobacilli was calculated as above indicated.

### Dynamic Simulation of the Child Gut Microbiota Supplemented with the Probiotic Candidate in the Food Carrier

Food-grade biomass of the selected strain (*L. plantarum* CECT 9435) was produced in ADM Biopolis (Valencia, Spain), which was supplemented with maltodextrin (67% bacterial dry matter, w/w) before lyophilization. Finally, individual sachets containing 3 g of lyophilized powder, having around 8 log CFU of viable bacteria per gram, were prepared.

The BFBL gut model is a four-stage reactors system intended to simulate in vitro the small intestine (SI) and the microbial conditions of three regions (R1, R2, R3) of the human colon [23]. At the beginning of the experiment, the colon reactors were all simultaneously inoculated with the same faecal sample from a pooled sample of five healthy children (aged 4–6 years) no receiving regular probiotic supplementation and prepared as described by Aguirre et al. [24]. The study was approved by the CSIC Committee of Ethics (protocol code 010/2018). The nutritive medium used for the development of colonic microbiota was based on previous studies [25] and adapted from the media developed by Cinquin et al. [26] that included casein (1 g/L), Tween 80 (1 g/L), lactose (0.5 g/L), MgSO_4_·7H_2_O /0.01 g/L) and haemin (0.001 g/L). The inoculated reactors were incubated overnight in batch conditions at 37 °C and continuously flushed with nitrogen. The pH in the colonic reactors was controlled by addition of 0.5 M NaOH and 0.5 M HCl to keep values of 5.7 ± 0.2 in R1, 6.3 ± 0.2 in R2 and 6.8 ± 0.2 in R3. The stabilization of the microbial community until steady-state conditions in the three colon reactors was achieved by feeding three times a day (morning, afternoon and night; during 2 weeks) the SI unit with the nutritive medium (pH 2) mixed with pancreatic juice [25], but reducing the Oxgall bile salts content to 3 g/L. After 2 h of digestion at 37 °C, the whole content of the SI unit was automatically transferred to the R1 colon compartment at a flow rate of 5 mL/min. The transfer of colonic content between the R1, R2, and R3 reactors was controlled with level sensors that keep their volumes at 200, 300, and 250 mL, respectively.

During the probiotic test period, lyophilised *L. plantarum* CECT 9435 was administered into the SI reactor at the morning feeding cycle of the Simulator BFBL and during 5 consecutive days by adding the bacteria from one sachet (previously washed with sterile water to remove the maltodextrin). In addition, 0.03 g skimmed milk and 0.05 g Incaparina®, to simulate the children nutritional intervention of the ProInfant project, were also added. The probiotic test period was followed by one-week wash-out period. During the experimental set up, samples were collected every 24 h from the three colonic reactors, as well as from the SI during the probiotic test period, and centrifuged (10,000 rpm for 10 min at 4 °C). Pellets and supernatants were separately stored at − 20 °C until further analysis. Each experiment was repeated thrice.

#### Microbiological Analyses

Samples in the reactors (R1, R2, R3) at the end of the first and second weeks of stabilization (S1, S2), the probiotic test period (P) and after one-week wash-out period (w/o) were selected to extract bacterial genomic DNA from the pellet according to the manufacturer’s instruction of the EZNA® Bacterial DNA Kit (Omega Biotek). Microbial cells were firstly lysed by mechanical disruption with glass beads (0.1 mm diameter zirconia/silica) (Sigma), using a FastPrep equipment for mechanical lysis (Bio 101 FastPrep FP120, Savant Instruments). DNA quantity were measured using a Nanodrop and then stored at − 20 °C for further analysis. Composition of the microbiota during the experiment was analysed by amplicon-based metagenomic-sequencing of the 16S rDNA V3-V4 region, performed by Novogen (Cambridge, UK), on a paired-end Illumina platform to generate 250 bp paired-end raw reads. Sequences with ≥ 97% similarity were assigned to the same Operational Taxonomic Unit (OTU). Total *Lactobacillus* spp. and specific *L. plantarum* CECT 9435 counts were performed by qPCR using SYBR green methodology in a ViiA7 System (Life Technologies) and including the genus-specific primers LabF362 and Lab-667-R [27] and the CECT 9435-specific primers Lp118B1For (5′-CTCAAGCGCACCACCCACCAC-3′) and Lp118B1Rev (5′-GCTCCATAACCGTTGCATAGACAG-3′). Primers were selected in the contig 0118 from the *L. plantarum* CECT 9435 genome sequence (GenBank: CAADHQ010000118.1) using the DNASTAR Primer Design Software (Lasergene 17).

#### Analysis of SCFA and Ammonium

Samples from the R1, R2 and R3 compartments were processed and analysed for SCFA content by HPLC as described earlier [25]. Calibration curves of acetic, propionic, butyric, formic, succinic and lactic acids were built up in the range concentration of 1 to 100 mM. Ammonium was determined using the Nessler’s reagent (Sigma) as previously described [28].

### Data Analyses

Results were expressed as media ± standard deviation (SD). One-way analysis of variance (ANOVA) and Tukey’s multiple comparison tests were performed to determine differences between treatment groups, employing SPSS Statistics for Windows, version 29.0 (IBM Corporation, Armonk, NY, USA). The significance level of statistical tests was set to *p* < 0.05. Principal Component Analysis (PCA) scatter plot was performed using STATISTICA program for Windows, version 7.1 (Statsoft Inc., Tulsa, OK, USA). Heatmap plot was performed applying the ClustVis webtool (https://biit.cs.ut.ee/clustvis/, accessed on 25 January 2024) [29].

## Results

### Selection of *L. plantarum* CECT 9435 as the Probiotic Candidate

**Fig. 1:**
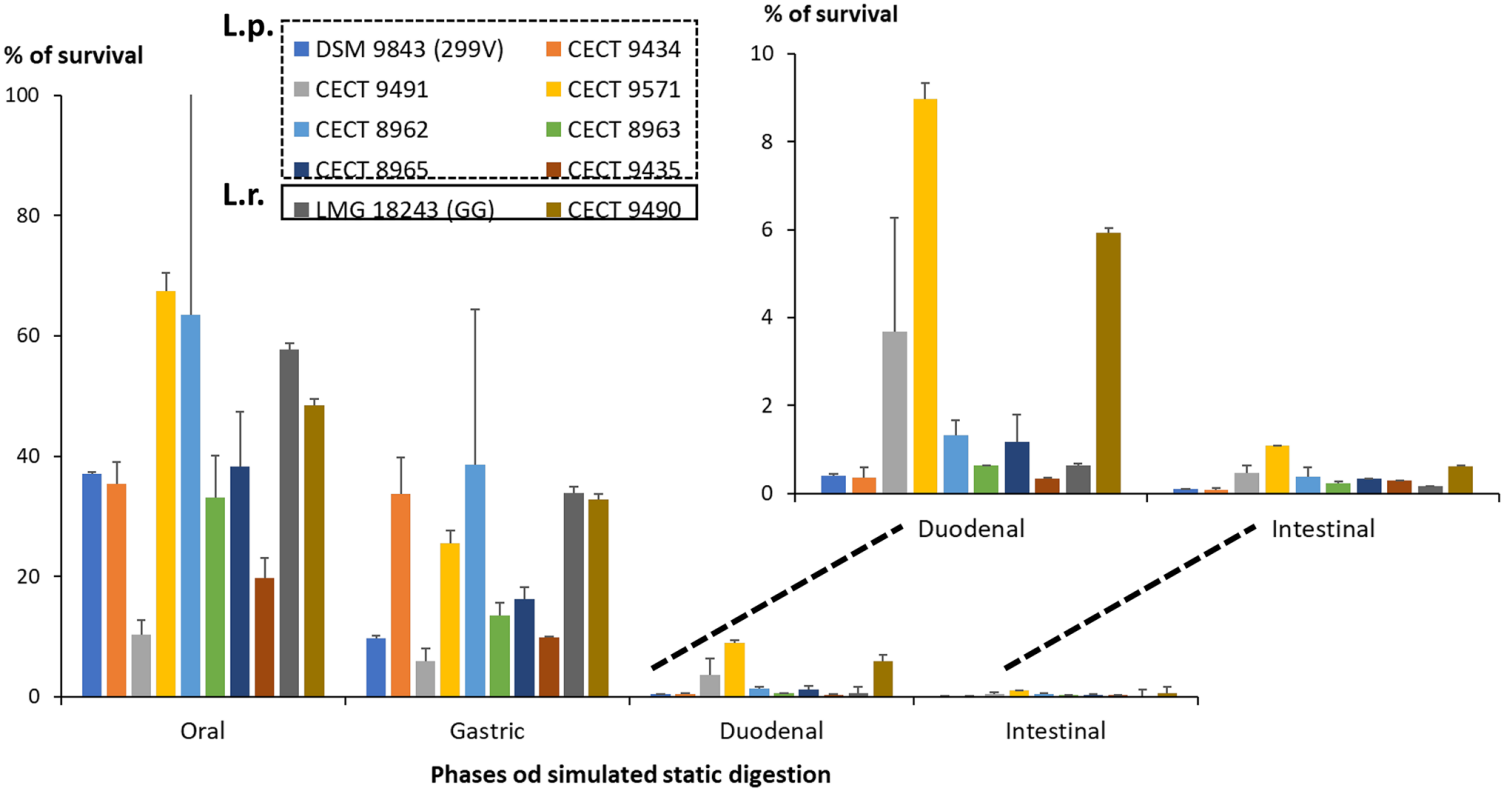
Percentage of survival of the eight lactobacilli isolated from Andean traditional foods included in a food-matrix after submitted to a sequential simulated static digestion (oral, gastric, duodenal and intestinal phases). The strains *Lactiplantibacillus plantarum* DSM 9843 (commercial brand: 299 V) and *Lacticaseibacillus rhamn*osus LMG 18243 (commercial brand: GG) were used as probiotics of reference. The percentage of survival was calculated as: 100*(CFU/mL of viable counts in each phase/CFU/mL of viable bacteria in the food suspension). See supplementary Table S1 for the data set

The selection of the probiotic candidate amongst the eight strains under study, to be tested in the BFBL gut model inoculated with child microbiota, was achieved by evaluating their survival to digestion, i.e. gastrointestinal passage, using a food-matrix as carrier. Besides, their adhesion capacity to the intestinal epithelium and the ability to compete with *E. coli* to in vitro adhere to this niche was evaluated upon the cell line HT29. Both features will be of special relevance for a probiotic candidate able to reduce the occurrence and/or frequency of diarrhoea associated to malnutrition in children. For that, the lactobacilli strains suspended in an Incaparina-like food matrix were sequentially submitted to a simulated static digestion. The behaviour under the four digestion phases was highly dependent on the strain (Fig. 1, Table S2). In general, the oral, and in less extent, the gastric challenges were better tolerated by all strains, whereas a drastic reduction in viability was observed when bile salts and pancreatin were added (duodenal and intestinal phases). The presence of these compounds at high concentrations (10 mM bile salts and 800 U pancreatin) even for a short time (10 min) reduced the survival of the strains below 4%, with the exception of the strains *L. plantarum* CECT 9571 and *L. rhamnosus* CECT 9490. This reduction was also observed for the probiotics of reference of these two species 299 V and GG, respectively. The duodenal digesta was diluted to reach about 3.3 mM bile salts and 267 U of pancreatin in the intestinal phase, which was prolonged for 2 h, but lactobacilli survival continued dropping in all case with values ranging from 0.09 to 1.08% (Table S2). However, it is worth noting that the number of viable bacteria were, especially in the case of *L. plantarum* strains, still higher than 1 × 10^7^ CFU/mL (Table S2), thus being on the levels of the recommended dose intake.

**Fig. 2:**
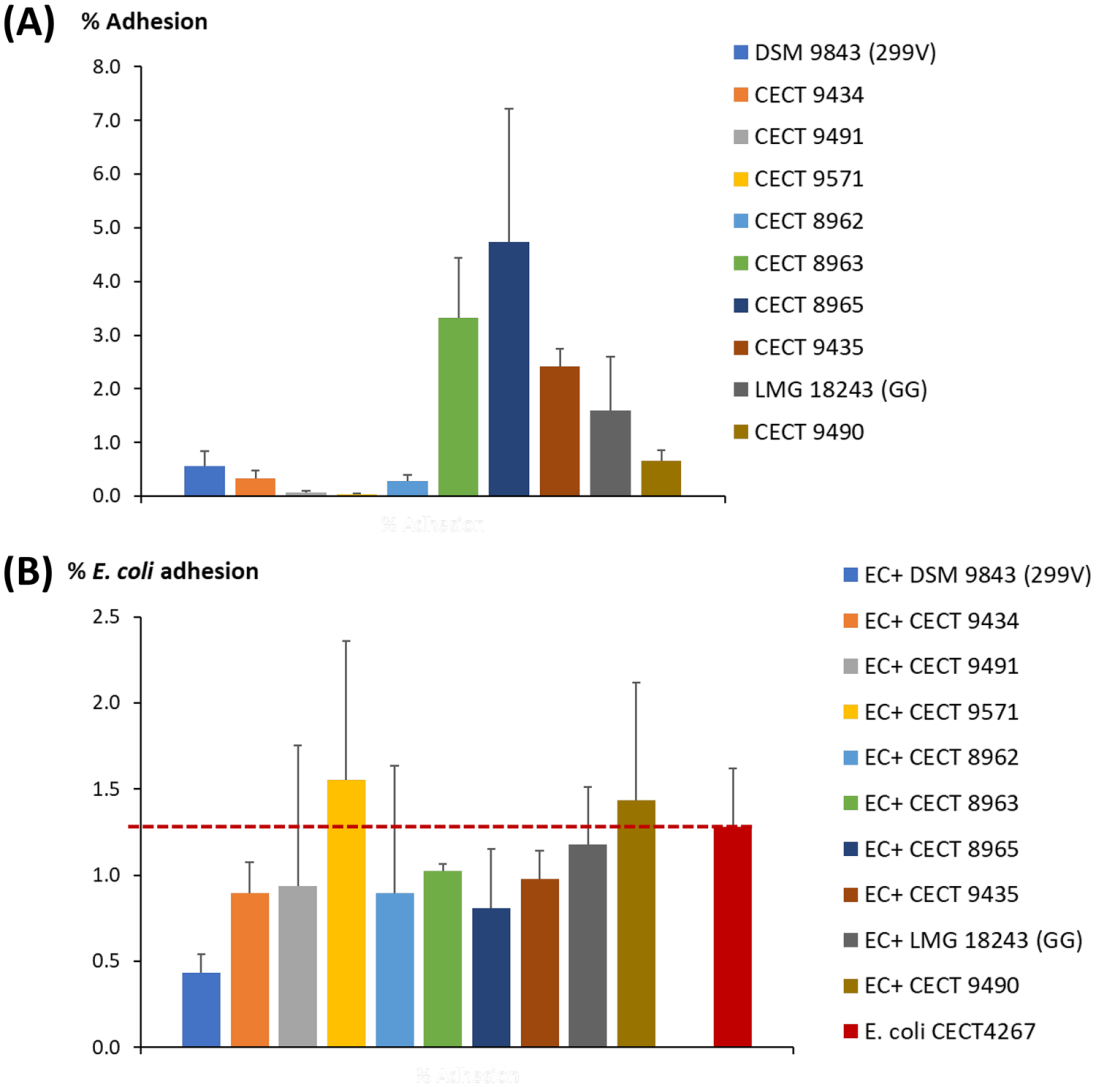
Adhesion of eight lactobacilli isolated from Andean traditional foods to the intestinal epithelial cell-line HT29; lactobacilli counts were made on agar-MRS (**A**). Adhesion of *E. coli* CECT 4267 to the cell-line HT29 in the absence (red bar) and in the presence (competition) of the lactobacilli; counts of *E. coli* were made on VRBA medium (**B**). The percentage of adhesion was calculated as follows: 100*(CFU/mL of adhered bacteria)/(CFU/mL of added bacteria). See supplementary Table S2 for data set

The ability of the lactobacilli to adhere to HT29 monolayers also revealed, as it could be expected, a high dependence on the strain tested (Fig. 2A). In this case, three *L. plantarum* strains showed a percentage of adhesion higher than the two reference probiotics: 3.32 ± 1.13% for CECT 8963, 4.74 ± 2.48% for CECT 8965 and 2.41 ± 0.34% for CECT 9435 (Table S3A). This feature is of special relevance for the antagonism against pathogens that could be involved in intestinal infections causing diarrhoea. Then, in a step further, the capability to compete with an enteropathogenic *E. coli* strain was tested. The three former strains, together with CECT 9434 and the probiotic of reference 299 V, were able to reduce the adhesion of *E. coli* CECT 4267 to HT29 monolayers (Fig. 2B). In this case, the average (± SD) adhesion values of the pathogen in the presence of the *L. plantarum* strains was below of that obtained when *E. coli* was added alone (Fig. 2B, Table S3B), the five mentioned strains being those showing higher reproducibility (lower coefficient of variation). The highest reduction in the adhesion of *E. coli* to HT29 was showed by probiotic 299 V (about 66%), followed by 37% of CECT 8965, 30% of CECT 9434, 24% of CECT 9435 and 20% of CECT 8963.

Finally, on the view of these results, three *L. plantarum* candidates were selected: CECT 8963, CECT 8965 and CECT 9435. They were sent to ADM-Biopolis, where additional criteria based on the highest biomass yield and better viability after lyophilization, were applied to finally chose the strain *L. plantarum* CECT 9435. The strain was food-grade produced at pilot scale to prepare ready-to-use mono-dose sachets for this study about its performance in the child intestinal microbiota stabilized in the BFBL dynamic gut model.

### Stabilization of Children Microbiota in the BFBL Dynamic Gut Model

The BFBL gut model was instrumental in this study to simulate the children intestinal microbiota. In comparison to previous studies with adult microbiota [23, 25], the experiment design included the reduction of the reactor volumes, which lowered the residence time, and the bile salt content of the pancreatic juice, as suggested by other authors [30]. Also, the basal nutrient medium was supplemented with casein, lactose and tween 80, plus haemin as an iron source [26]. The BFBL gut model was inoculated with a pooled faecal sample obtained from five children aged from four to six years. Samples were analysed after 1 and 2 weeks of microbial stabilization in the colonic reactors. Both, the number of observed taxa and the diversity index values (Table S4), were higher in the inoculum than in the microbiota developed in the three reactors and they trend to decrease with time during the two weeks of stabilization. Over 70% of taxa with abundances higher than 0.01% found in the inoculum (InInf) were detected in the colon reactors (results not shown). Main InInf taxa not found in the reactors (comprising 1% total abundance in the inoculum) were from the order *Clostridiales*, specifically the families *Ruminococcaceae* and *Lachnospiraceae*.

**Fig. 3:**
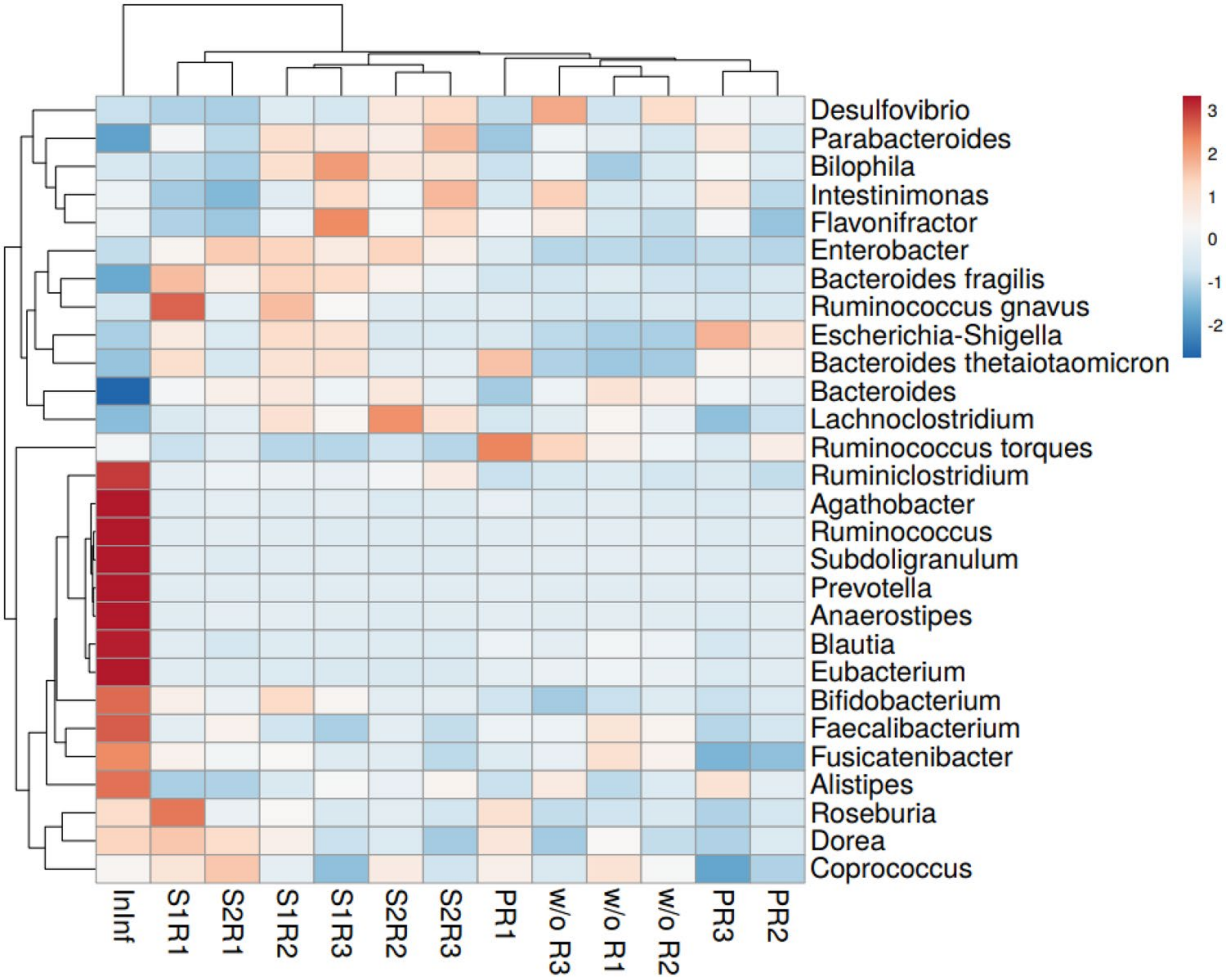
Heatmap of the genus-level hierarchical clustering of the microbial community (abundance > 0.1%) in the pooled child faecal microbiota (InInf) and in the reactors (R1, R2, R3) during the first and second weeks of stabilization (S1, S2), at the probiotic test period (P) and after one-week wash-out period (w/o)

Figure 3 shows the heatmap with the distribution and variability of the bacterial genera found with abundances higher than 0.1% in the children inoculum (InInf). Main genera in the children pool were *Bifidobacterium* (17.6%), which decreased by time during stabilization in the BFBL gut model and was equally distributed in the three reactors (Fig. 3, Table S5). *Faecalibacterium* (8.7% in the inoculum) also decreased from reactor R1 to R3 but increased by time. Amongst the genera that developed higher abundances in the BFBL gut model than those in the inoculum, there were *Bacteroides*, *Parabacteroides* and *Enterobacter* (Fig. 3, Table S5). Clustering PCA grouped by reactor the genera with abundances higher than 0.1% (Fig. S1). On the other hand, results shown in Fig. 3 denoted that at the end of the first week of stabilization, the microbiota developed in the reactors was closer to the pooled child microbiota InInf than at the end of the second week of stabilization.

Regarding lactobacilli content of the child microbiota, considering the 16S rRNA amplicon sequencing results as a combination of the 25 renamed genera previously ascribed to the genus *Lactobacillus*, sequencing results showed low abundance in the children inoculum (0.009%; Table S5). During stabilization in the BFBL gut model, results indicated higher values in the proximal reactor (R1) than in R2 and R3 at the end of the stabilization period (S2) of the child microbiota in the BFBL gut model (Table 2) and a general trend to decrease with time. The same trend was observed with the taxon *L. plantarum*. When the concentration of lactobacilli was analysed by qPCR using the genus-specific primers, the values were not significantly different between reactors and time of the study. As expected, the strain-specific qPCR analysis revealed that *L. plantarum* CECT 9435 was under the detection limit (2.26 ± 0.28 log copy/mL) in all reactors during the stabilization period.

**Table 2:** Lactobacilli abundance (%, and SD in parenthesis) and qPCR counts (log copy/mL and SD) in the reactors (R1, R2, R3) during the first and second weeks of stabilization (S1, S2), at the probiotic test period (P) and after one-week wash-out period (w/o)

	**R1**				**R2**				**R3**			
	**S1**	**S2**	**P**	**w/o**	**S1**	**S2**	**P**	**w/o**	**S1**	**S2**	**P**	**w/o**
Lactobacilli (abundance %)	0.015^a,b^ (0.010)	0.013^a,b^ (0.008)	0.013^a,b^ (0.007)	0.003^a^ (0.000)	0.008^a,b^ (0.007)	0.003^a^ (0.002)	0.018^b^ (0.002)	0.005^a^ (0.004)	0.008^a,b^ (0.001)	0.002^a^ (0.002)	0.011^a,b^ (0.001)	0.006^a,b^ (0.002)
*L. plantarum* (abundance %)	0.009^a,b^ (0.008)	0.008^a,b^ (0.008)	0.012^a,b^ (0.008)	0.001^a^ (0.001)	0.006^a,b^ (0.007)	0.001^a^ (0.001)	0.010^a,b^ (0.006)	0.005^a^ (0.004)	0.006^a,b^ (0.001)	0.001^a^ (0.002)	0.007^a,b^ (0.003)	0.006^a,b^ (0.002)
Lactobacilli (log copy/mL)	4.083^a,b^ (0.502)	4.227^a,b^ (0.725)	5.203^b,c^ (0.202)	3.987^a^ (0.421)	3.730^a^ (0.437)	4.200^a,b^ (0.510)	5.400^c^ (0.144)	4.030^a^ (0.294)	4.057^a,b^ (0.146)	4.350^a,b^ (0.540)	5.343^c^ (0.136)	4.170^a,b^ (0.312)
*L. plantarum* CECT 9435 (log copy/mL)	nd	nd	5.197^a^ (0.108)	nd	nd	nd	5.377^a,b^ (0.032)	nd	nd	nd	5.463^b^ (0.186)	nd

Different letters (a, b, c) in the same row indicate significant differences (P < 0.05) between reactors and time periods using one-way ANOVA analysis. nd, under detection limit (2.26 ± 0.28 log copy/mL)

### Supplementation of Child Gut Model with the Probiotic Candidate

The daily supplementation of *L. plantarum* CECT 9435 for five days in the BFBL gut model showed, at the end of the intervention (P), a trend to increase the abundance of *L. plantarum* in all the reactors. The differences were significant when analysed by qPCR both using genus-specific primers and strain-specific primers (Table 2). The strain-specificity of the primers was confirmed with DNA from several lactobacilli and *L. plantarum* strains and human faecal microbiota (results not shown). During probiotic supplementation to the BFBL gut model, the lactobacilli counts increased one log to reach over 5 log copy/mL in the three reactors. The increase of lactobacilli corresponded with the values of the qPCR quantification of the probiotic (Table 2). As observed for the viability of the probiotic candidates under simulated static digestion (Table S2), survival of *L. plantarum* CECT 9435 in the colonic conditions was over 1 × 10^7^ counts per reactor. On the other hand, lactobacilli qPCR counts, targeting both the genus and the CECT 9435 strain, returned at the end of the wash out (w/o) period to the counts of the stabilization period (S1 and S2 stages)*,* indicating low colonization capacity of *L. plantarum* CECT 9435 in the child microbiota developed in the BFBL gut model.

Other than the mentioned changes in lactobacilli qPCR counts, the supplementation of the probiotic strain could not be associated to substantial changes in the abundance of the main genera stabilized in the children BFBL gut model pool (Fig. 3, Table S5). During the probiotic supplementation, we observed a trend to decrease the abundance of some proteobacteria genera such as *Enterobacter*, *Desulfovibrio*, and *Bilophila*, and to increase the *Escherichia*-*Shigella* group. Some of these observed changes were restored during the wash out period (Table S5).

### Formation of SCFA and Ammonium

The stabilization of the child microbiota in the BFBL gut model was characterized by the formation of acetic, propionic and butyric acids as the main SCFA. The concentration of acetic and propionic acids increased from reactors R1 to R3, and there were not significant differences between the end of weeks 1 (stage S1) and 2 (stage S2) of stabilization (Table 3). However, the butyrate concentration in R3 decreased during stabilization to lower values than the expected from the accumulation of SCFA in the distal compartments observed for three-stage culture reactors without absorption steps. The ammonium content also increased from R1 to R3, showing not significant differences between the first and second week of stabilization.

**Table 3:** Mean and SD (in parenthesis) of microbial metabolites (mM) produced in the reactors (R1, R2, R3) during the first and second weeks of stabilization (S1, S2), at the probiotic test period (P) and after one-week wash-out period (w/o)

	**R1**				**R2**				**R3**			
**Metabolite**	**S1**	**S2**	**P**	**w/o**	**S1**	**S2**	**P**	**w/o**	**S1**	**S2**	**P**	**w/o**
Acetate	28.07^a^ (4.47)	31.15^a,b^ (1.91)	30.37^a,b^ (7.75)	32.04^a,b^ (3.09)	41.25^a,b^ (3.69)	46.02^b,c^ (2.11)	54.08^b,c^ (7.39)	49.28^b,c^ (3.19)	58.46^c,d^ (11.77)	63.74^c,d^ (0.13)	72.49^d^ (9.35)	70.46^d^ (8.20)
Propionate	6.11^a^ (0.91)	6.48^a^ (1.06)	5.42^a^ (0.20)	6.22^a^ (0.28)	8.56^a,b^ (0.79)	9.34^b^ (0.89)	8.84^a,b^ (0.73)	8.11^a,b^ (0.85)	10.64^b^ (1.64)	11.36^b^ (0.59)	10.92^b^ (0.08)	10.05^b^ (0.90)
Butyrate	11.14^b,c^ (1.65)	12.04^b,c^ (2.42)	16.86^c^ (3.81)	12.92^b,c^ (0.87)	9.22^b,c^ (0.81)	10.53^b,c^ (3.05)	19.77^c^ (6.96)	13.24^b,c^ (2.23)	1.82^a^ (0.19)	3.40^a^ (1.31)	11.98^b,c^ (7.59)	7.48^b^ (3.65)
Ammonium	11.29^a^ (3.28)	12.70^a,b^ (3.17)	11.03^a^ (1.14)	12.13^a,b^ (1.38)	18.29^b,c^ (2.56)	18.65^b,c^ (2.23)	15.90^b,c^ (2.27)	16.44^b,c^ (0.12)	19.68^c^ (3.35)	20.37^c^ (2.12)	17.70^b,c^ (0.47)	16.12^b,c^ (0.54)

Different letters (a, b, c) in the same row indicate significant differences (P < 0.05) between reactors and time periods using one-way ANOVA analysis

The addition of *L. plantarum* CECT 9435 resulted in no significant changes (*p* > 0.05) of neither SCFA nor ammonium contents, although a trend to increase the formation of butyrate was observed (Table 3). In addition, the content of ammonium had a tendency to decrease during the probiotic treatment. The results indicate that the transient presence of the bacteria did not affect significantly the global metabolism of the microbiota.

## Discussion

The selected *L. plantarum* CECT 9435 (M9MG6-B2) is a riboflavin overproducing derivative from strain CECT 8965 (M9MG6) that was isolated from chicha, a traditional maize-based fermented beverage from Northwester Argentina [18] in the frame of the project MICROANDES. The parental strain CECT 8965 demonstrated an antimicrobial effect, it was sensitive to several antibiotics [31] and produced high amounts of riboflavin, also exhibiting good technological performance under different food related-stressors, similarly to its derivative CECT 9435 strain [32]. Additionally, the last one showed ability to survive under in vitro gastrointestinal challenges and high adhesion capacity to Caco-2 epithelial cells [33]. In the current work, these traits were confirmed with additional models. *L. plantarum* CECT 9435 included in a food matrix survived to the digestion in levels similar to those of the reference probiotic *L. plantarum* 299 V strain. Besides, it showed a good adhesion to HT29, a cell line composed of colonocytes and mucus-producing Goblet cells, with values higher than the reference probiotic. In addition, it fulfils the EFSA food safety requirements following assessment by genomic analysis [15], belonging to one of the lactobacilli species included in the QPS list [34]. Finally, the outstanding performance in *E. coli* competition for the adhesion to HT29 prompted the selection of *L. plantarum* CECT 9435 for study its performance in a dynamic gut model inoculated with child microbiota and feed the food carrier.

There are not many dynamic and multi-compartmental models simulating the child gut microbiota [35]. In this study, the BFBL gut model was adapted for holding child microbiota by decreasing the bile salt concentration and reactor residence time and adapting the nutritive medium as described by Cinquin et al. [26]. The homology and proportion of taxa, as well as the stabilization of SCFA concentrations in the BFBL gut model running after 8–10 days of the experiment set up, could be considered optimal for starting dietary interventional studies. At this period, the percentage of taxa representative of the child inoculum was high and it could avoid a significant reduction of the abundance of some bacterial populations, like *Bifidobacterium* that are considered the taxon representative of healthy children intestinal microbiota [36], or the overgrowth of taxa like *Bacteroides* and *Enterobacteriaceae*. An unexpected result during child microbiota stabilization was the low concentration of butyrate in the R3 stage of the colonic model, which was observed in all replicates and further studies (results not shown). However, this behaviour was not previously observed during adult microbiota stabilization in similar conditions [23, 25]. This could be due to the decreased abundance of butyrate producers observed in R3 during stabilization, including genera integrated in the *Lachnospiraceae* family, mainly *Dorea, Coprococcus* and *Roseburia*, and also lower values of *Faecalibacterium* from the family *Ruminococcaceae* (Table S5), which are characteristic butyrate-producing taxa [37]. Lower butyrate concentration in infant faeces than in adults has also been reported [35]. Increase of butyrate formation has been reported in the transition from infant to adult gut microbiota, which correlated with bacterial networks associated with butyrate-producers [38] and butyrate-consumers. Indeed, higher abundance of syntrophic bacteria (butyrate-degrading bacteria) *Bilophila* and *Desulfovibrio* could be observed in R3 (Table S5), which might also account for the butyrate decrease observed in the last stage of the child-gut model.

Regarding results obtained after the intervention with the probiotic candidate *L. plantarum* CECT 9435, it showed a low colonization capability. This has been also observed with other *L. plantarum* of the same origin, such as the strain CECT 9434 (Table 1) harbouring the plasmid pRCR12 (with mCherry marker), in vitro tested in the BFBL gut model using an inoculum from adult microbiota [11]. Considering the age of the children used as faecal inoculum in the current study, in which a decline in the intake of milk and the incorporation of solid foods with increased amount of plant glycans (dietary fibre) could be expected, the data obtained suggest that the gut microbiota diversity of pre-school and school-age children is similar to that of adults in terms of their global composition, but still showing higher *Bifidobacterium* levels than in adults as already documented [39]. In general, resistance of intestinal microbiota colonization by probiotics increases with community richness and diversity [10, 40]. Besides, the concentration of lactobacilli in the child microbiota inoculum of our study was low, that could denote that this microbial niche was not favouring the residence of the strain *L. plantarum* CECT 9435 [41]*.*

On the other hand, the good adhesion percentages of *L. plantarum* CECT 9435 to epithelial HT29 monolayer observed, as well as to other cellular models [33], could facilitate its potential benefits for vitamin production, barrier functions, etc., based on its increasing numbers during gut transit. Efficient use of *L. plantarum* as probiotic has been demonstrated when used to improve the nutritional status of undernourished children [42], on clinical symptoms, including diarrhoea, in children with rotaviral enteritis [43], and to reduce the severity of common cold infections in children attending day care [44], amongst others [45].

## Supplementary Information

 Below is the link to the electronic supplementary material.

Supplementary file1 (DOCX 215 kb)

## Data Availability

Additional data will be made available on request.
