# Supplementary material for: Probiotic Characteristics of Lactiplantibacillus plantarum CECT 9435 and Its Survival and Competitive Properties Under Simulated Conditions of the Child Gut Microbiota
Source: Probiotics Antimicrob Proteins. 2024 May 3;17(4):1839–50. doi: 10.1007/s12602-024-10280-w (PMC12405041; doi:10.1007/s12602-024-10280-w)

**SUPPLEMENTARY MATERIAL**

**Probiotic characteristics of *Lactiplantibacillus plantarum* CECT 9435 and its survival and competitive properties under simulated conditions of the child gut microbiota**

Requena, T.**^1^**, Martínez-Cuesta, M.C.**^1^**, Aznar, R.**^2^**, Mohedano, M.L.^3^, López, P.**^3^**, Ruas-Madiedo, P.**^4^**

**^1^**Instituto de Investigación en Ciencias de la Alimentación (CIAL), CSIC, Department of Food Biotechnology and Microbiology, BFBL group. Nicolas Cabrera 9, 28049, Madrid, Spain

^2^Department of Microbiology and Ecology and Spanish Collection of Type Cultures (CECT), University of Valencia, Valencia, Spain

^3^Department of Microorganisms and Plant Biotechnology, Margarita Salas Center for Biological Research (CIB)-Consejo Superior de Investigaciones Científicas (CSIC), Madrid, Spain

**^4^**Instituto de Productos Lácteos de Asturias (IPLA), CSIC, MicroHealth group. Paseo Río Linares s/n, 33300, Villaviciosa, Asturias, Spain

**Table S1**. Values of pH of the food-bacterial suspensions along the static gastrointestinal simulation (oral, gastric, duodenal and intestinal) steps. The initial sample is the bacterial suspension prepared in the food matrix.

|  |  | **pH** (mean ± SD) | | | | |
| --- | --- | --- | --- | --- | --- | --- |
|  |  | **Initial** | **Oral** | **Gastric** | **Duodenal** | **Intestinal** |
| *L. rhamnosus** | CECT 9490 | 6.00±0.01 | 6.66±0.03 | 2.93±0.04 | 7.61±0.00 | 8.70±0.05 |
|  | LMG 18243 | 5.97±0.02 | 6.46±0.06 | 3.27±0.06 | 7.03±0.06 | 8.57±0.02 |
| *L. plantarum** | CECT 8962 | 5.84±0.01 | 6.29±0.05 | 2.91±0.04 | 7.47±0.27 | 8.46±0.11 |
|  | CECT 8963 | 5.91±0.04 | 6.40±0.00 | 2.84±0.04 | 6.99±0.04 | 8.10±0.11 |
|  | CECT 8965 | 5.80±0.01 | 6.46±0.27 | 3.10±0.01 | 7.09±0.09 | 7.94±0.06 |
|  | CECT 9434 | 6.02±0.01 | 5.80±0.02 | 2.86±0.06 | 7.09±0.04 | 8.70±0.14 |
|  | CECT 9435 | 5.40±0.00 | 6.09±0.01 | 2.98±0.11 | 7.39±0.06 | 8.39±0.03 |
|  | CECT 9491 | 5.06±0.01 | 6.44±0.00 | 3.11±0.05 | 7.48±0.11 | 8.40±0.11 |
|  | CECT 9571 | 5.97±0.02 | 5.79±0.08 | 2.95±0.06 | 7.34±0.08 | 8.30±0.11 |
|  | DSM 9843 | 5.80±0.02 | 5.90±0.13 | 3.16±0.06 | 7.47±0.07 | 8.29±0.03 |

**Lactiplantibacillus plantarum* and *Lacticaseibacillus rhamnosus*

**Table S2**. Counts in agar-MRS of lactobacilli suspensions in a food carrier (initial counts) after being sequentially submitted to a simulated static digestion following four sequential phases: oral, gastric, duodenal and intestinal. The final survival [(CFU/ml of intestinal phase) / (CFU/ml of initial bacterial suspensions in food)], expressed as percentage, is also included. The values of final survival (intestinal phase) are those depicted in Figure 1. The grey-shadow strains were those selected to evaluate the technological performance in ADM-Biopolis, and the blue one the final selected probiotic candidate.

|  |  | **Counts** (CFU/ml) | | | | | **Final** |
| --- | --- | --- | --- | --- | --- | --- | --- |
| Species | Strains | **Initial** | **Oral** | **Gastric** | **Duodenal** | **Intestinal** | **Survival (%)** |
| *L. plantarum** | **DSM 9843** CECT 9434 CECT 9491 CECT 9571 CECT 8962 CECT 8963 CECT 8965 **CECT 9435** | 1.7E+10 ±0.14E+10 5.5E+09 ±0.78E+09 6.0E+09 ±1.56E+09 2.4E+09 ±0.21E+09 3.5E+09 ±2.69E+09 6.1E+09 ±1.48E+09 4.3E+09 ±0.64E+09 4.1E+09 ±0.92E+09 | 6.3E+09 ±0.57E+09 2.0E+09 ±0.07E+09 6.0E+08 ±0.07E+08 1.7E+09 ±0.07E+09 1.7E+09 ±0.40E+09 2.0E+09 ±0.07E+09 1.6E+09 ±0.14E+09 7.9E+08 ±0.50E+08 | 1.7E+09 ±0.07E+09 1.9E+09 ±0.07E+09 3.4E+08 ±0.35E+08 6.3E+08 ±1.06E+08 1.1E+09 ±0.13E+09 8.1E+08 ±0.78E+08 7.0E+08 ±1.91E+08 4.0E+08 ±0.85E+08 | 6.8E+07 ±1.13E+07 1.0E+07 ±0.99E+07 2.0E+08 ±0.99E+08 2.2E+08 ±0.28E+08 4.2E+07 ±2.22E+07 3.9E+07 ±0.92E+07 4.8E+07 ±1.84E+07 1.3E+07 ±0.14E+07 | 1.8E+07 ±0.14E+07 4.6E+06 ±0.92E+06 2.7E+07 ±0.35E+07 2.7E+07 ±0.21E+07 1.0E+07 ±0.26E+07 1.4E+07 ±0.14E+07 1.4E+07 ±0.28E+07 1.2E+07 ±0.21E+07 | 0.11±0.01 0.09±0.03 0.46±0.18 1.08±0.01 0.37±0.21 0.24±0.03 0.33±0.02 0.29±0.01 |
| *L. rhamnosus** | **LMG 18243** CECT 9490 | 1.1E+09 ±0.07E+09 1.5E+09 ±0.07E+09 | 6.1E+09 ±1.56E+08 7.1E+08 ±1.20E+08 | 3.6E+08 ±0.07E+08 4.7E+08 ±1.70E+08 | 6.8E+06 ±0.92E+06 8.6E+07 ±0.57E+07 | 1.8E+06 ±0.07E+06 8.9E+06 ±0.79E+06 | 0.17±0.01 0.61±0.02 |

**Lactiplantibacillus plantarum* and *Lacticaseibacillus rhamnosus*

**Table S3**. Counts (CFU/ml) in agar-MRS of lactobacilli added and adhered to the intestinal cell-line HT29 (A). Counts (CFUC/ml) in VRBA of *E. coli* CECT 4267 added and adhered to the intestinal cell-line HT29 in the absence and in the presence of the lactobacilli strains. The percentages of adhesion (CFU/ml of bacteria adhered/CFU of bacteria added) are those represented in Figure 2. The grey-shadow strains were those selected to evaluate the technological performance in ADM-Biopolis, and the blue one the final selected probiotic candidate.

|  |  | **Counts** (CFU/ml) | | | |  | |
| --- | --- | --- | --- | --- | --- | --- | --- |
| **(A)** | Strains | Bacteria **added** | Bacteria **adhered** | | | **Adhesion** (%) | |
| *L. plantarum** | **DSM 9843** CECT 9434 CECT 9491 CECT 9571 CECT 8962 CECT 8963 CECT 8965 **CECT 9435** | 6.0E+08±1.46E+08 3.0E+08±0.33E+08 5.8E+08±1.17E+08 3.6E+08±1.69E+08 2.8E+08±0.41E+08 1.6E+08±0.47E+08 9.9E+07±3.59E+07 1.1E+08±0.42E+08 | 3.3E+06±1.79E+06 9.5E+05±3.55E+05 3.8E+05±1.51E+05 1.3E+05±0.26E+05 7.7E+05±3.25E+05 5.6E+06±3.35E+06 4.1E+06±0.67E+06 2.5E+06±0.66E+06 | | | 0.55±0.29 0.33±0.14 0.07±0.03 0.04±0.01 0.28±0.11 3.32±1.13 4.74±2.48 2.41±0.34 | |
| *L. rhamnosus** | **LMG 18243** CECT 9490 | 1.1E+08±0.53E+08 5.9E+07±2.43E+07 | 1.7E+06±0.88E+06 3.9E+05±1.86E+05 | | | 1.60±0.85 0.66±0.19 | |
| **(B)** |  |  | |  |  | |  |
| *E. coli* | **CECT4267 (alone)** + **DSM 9843** + CECT 9434 + CECT 9491 + CECT 9571 + CECT 8962 + CECT 8963 + CECT 8965 + **CECT 9435** + **LMG 18243** + CECT 9490 | 4.2E+07±2.01E+07 | | 5.6E+05±3.26E+05 2.0E+05±1.24E+05 3.9E+05±2.17E+05 2.9E+05±1.17E+05 5.6E+05±2.19E+05 2.8E+05±0.79E+05 4.4E+05±1.92E+05 2.9E+05±0.54E+05 3.2E+05±1.55E+05 5.2E+05±3.48E+05 5.6E+05±3.54E+05 | 1.28±0.34 0.43±0.11 0.90±0.18 0.94±0.81 1.55±0.81 0.89±0.74 1.02±0.04 0.81±0.35 0.98±0.17 1.18±0.33 1.44±0.68 | |  |

**Lactiplantibacillus plantarum* and *Lacticaseibacillus rhamnosus*

**Table S4**. Alpha-diversity comparisons between the children microbiota pooled-inoculum (InInf), and the microbiota developed in the three reactors (R1, R2, R3) during the two weeks of stabilization (S1, S2), the supplementation with *Lactiplantibacillus plantarum* CECT 9435 (P) and the wash-out (w/o) period. Observed OTUs and Chao1 are species richness estimators, the Shannon index is a species evenness estimator, and the Simpson index is a dominance species estimator.

|  | **Observed_OTUs** | **Chao1** | **Shannon** | **Simpson** |
| --- | --- | --- | --- | --- |
| InInf | 425 ± 45 | 451.82 ± 53.58 | 5.57 ± 0.16 | 0.95 ± 0.01 |
| S1R1 | 299 ± 45 | 324.51 ± 62.97 | 4.31 ± 0.28 | 0.87 ± 0.04 |
| S2R1 | 312 ± 27 | 359.27 ± 38.25 | 4.22 ± 0.53 | 0.85 ± 0.09 |
| S1R2 | 302 ± 26 | 329.02 ± 25.85 | 4.39 ± 0.23 | 0.87 ± 0.04 |
| S2R2 | 284 ± 1 | 314.35 ± 10.98 | 4.06 ± 0.41 | 0.83 ± 0.07 |
| S1R3 | 310 ± 48 | 347.00 ± 43.96 | 4.07 ± 0.34 | 0.83 ± 0.04 |
| S2R3 | 292 ± 10 | 321.09 ± 23.62 | 3.80 ± 0.63 | 0.77 ± 0.13 |
| PR1 | 300 ± 36 | 362.09 ± 54.04 | 3.94 ± 0.06 | 0.84 ± 0.04 |
| PR2 | 297 ± 4 | 333.26 ± 6.73 | 3.84 ± 0.19 | 0.82 ± 0.03 |
| PR3 | 275 ± 23 | 319.95 ± 25.70 | 3.79 ± 0.63 | 0.80 ± 0.03 |
| w/oR1 | 274 ± 48 | 287.16 ± 46.66 | 4.09 ± 0.16 | 0.84 ± 0.02 |
| w/oR2 | 310 ± 13 | 335.45 ± 24.46 | 3.98 ± 0.54 | 0.82 ± 0.09 |
| w/oR3 | 292 ± 16 | 305.47 ± 18.57 | 3.92 ± 0.57 | 0.80 ± 0.11 |

**Table S5**. Abundance (%) of genera, species or groups higher than 0.1% and *Lactobacillus* in the inoculum (InInf) and samples from R1, R2, R3 at the end of the first (S1) and second week (S2) of stabilization in the BFBL gut model, at the end of the probiotic supplementation (PR1, PR2, PR3), and at the end of the wash-out (w/o) period.

| **Genus / group** | **InInf** | **S1R1** | **S2R1** | **S1R2** | **S2R2** | **S1R3** | **S2R3** | **PR1** | **PR2** | **PR3** | **w/oR1** | **w/oR2** | **w/oR3** |
| --- | --- | --- | --- | --- | --- | --- | --- | --- | --- | --- | --- | --- | --- |
| *Agathobacter* | 2.844 | 0.167 | 0.246 | 0.130 | 0.077 | 0.213 | 0.115 | 0.293 | 0.187 | 0.034 | 0.183 | 0.100 | 0.087 |
| *Alistipes* | 0.902 | 0.023 | 0.032 | 0.166 | 0.259 | 0.339 | 0.386 | 0.086 | 0.229 | 0.521 | 0.063 | 0.188 | 0.462 |
| *Anaerostipes* | 2.113 | 0.122 | 0.186 | 0.145 | 0.101 | 0.124 | 0.116 | 0.156 | 0.132 | 0.050 | 0.146 | 0.128 | 0.111 |
| *Bacteroides* | 5.108 | 11.071 | 11.860 | 12.365 | 12.301 | 10.915 | 10.359 | 8.379 | 10.345 | 10.897 | 12.766 | 12.009 | 10.703 |
| *Bacteroides fragilis* | 2.419 | 9.868 | 7.447 | 9.189 | 7.219 | 8.927 | 6.080 | 4.752 | 4.960 | 4.459 | 5.426 | 4.653 | 4.673 |
| *Bacteroides thetaiotaomicron* | 0.829 | 5.381 | 2.294 | 5.200 | 2.894 | 5.301 | 2.982 | 6.272 | 4.096 | 4.046 | 0.968 | 1.101 | 1.310 |
| *Bifidobacterium* | 17.608 | 11.298 | 9.415 | 13.376 | 8.908 | 10.705 | 8.966 | 7.486 | 8.278 | 7.130 | 7.160 | 8.471 | 6.100 |
| *Bilophila* | 0.219 | 0.167 | 0.122 | 0.527 | 0.481 | 0.704 | 0.491 | 0.173 | 0.240 | 0.372 | 0.109 | 0.221 | 0.335 |
| *Blautia* | 4.902 | 0.990 | 0.667 | 0.903 | 0.937 | 0.881 | 0.853 | 1.330 | 0.971 | 0.615 | 1.449 | 1.295 | 1.051 |
| *Citrobacter* | 4.902 | 0.990 | 0.667 | 0.903 | 0.937 | 0.881 | 0.853 | 1.330 | 0.971 | 0.615 | 1.449 | 1.295 | 1.051 |
| *Coprococcus* | 1.186 | 1.388 | 1.629 | 0.978 | 1.307 | 0.544 | 0.784 | 1.260 | 0.663 | 0.393 | 1.424 | 1.150 | 0.876 |
| *Desulfovibrio* | 0.108 | 0.049 | 0.040 | 0.192 | 0.450 | 0.138 | 0.539 | 0.095 | 0.264 | 0.313 | 0.117 | 0.528 | 0.684 |
| *Dorea* | 2.048 | 2.145 | 1.953 | 1.658 | 1.193 | 1.016 | 0.837 | 1.806 | 1.202 | 0.904 | 1.563 | 0.991 | 0.836 |
| *Enterobacter* | 0.186 | 1.447 | 2.442 | 2.329 | 2.352 | 1.724 | 1.574 | 0.662 | 0.100 | 0.175 | 0.117 | 0.057 | 0.054 |
| *Escherichia-Shigella* | 0.393 | 1.910 | 0.954 | 2.231 | 0.904 | 2.163 | 0.947 | 0.903 | 2.113 | 2.754 | 0.366 | 0.385 | 0.528 |
| *Eubacterium* | 0.724 | 0.073 | 0.062 | 0.059 | 0.043 | 0.045 | 0.044 | 0.103 | 0.081 | 0.049 | 0.145 | 0.125 | 0.119 |
| *Faecalibacterium* | 8.696 | 3.098 | 4.422 | 2.152 | 3.033 | 1.393 | 1.924 | 3.469 | 2.357 | 1.669 | 5.245 | 4.382 | 3.508 |
| *Flavonifractor* | 0.131 | 0.084 | 0.077 | 0.129 | 0.138 | 0.224 | 0.177 | 0.136 | 0.072 | 0.137 | 0.105 | 0.090 | 0.153 |
| *Fusicatenibacter* | 2.221 | 1.480 | 1.329 | 1.407 | 1.143 | 1.124 | 0.887 | 1.120 | 0.725 | 0.655 | 1.707 | 1.473 | 1.229 |
| *Intestinimonas* | 0.186 | 0.091 | 0.066 | 0.165 | 0.198 | 0.286 | 0.331 | 0.140 | 0.112 | 0.252 | 0.144 | 0.152 | 0.303 |
| *Lachnoclostridium* | 0.814 | 1.021 | 1.080 | 1.365 | 1.623 | 1.214 | 1.353 | 0.994 | 0.958 | 0.828 | 1.220 | 1.120 | 1.066 |
| *Parabacteroides* | 0.655 | 1.758 | 1.147 | 2.248 | 1.990 | 2.136 | 2.549 | 0.992 | 1.346 | 2.091 | 1.519 | 1.345 | 1.702 |
| *Prevotella* | 2.007 | 0.008 | 0.017 | 0.013 | 0.004 | 0.008 | 0.007 | 0.024 | 0.015 | 0.000 | 0.009 | 0.007 | 0.010 |
| *Roseburia* | 0.251 | 0.382 | 0.116 | 0.154 | 0.097 | 0.075 | 0.053 | 0.237 | 0.061 | 0.013 | 0.046 | 0.073 | 0.032 |
| *Ruminiclostridium* | 0.396 | 0.095 | 0.112 | 0.107 | 0.127 | 0.101 | 0.174 | 0.035 | 0.028 | 0.067 | 0.078 | 0.048 | 0.057 |
| *Ruminococcus* | 4.340 | 0.146 | 0.202 | 0.162 | 0.120 | 0.117 | 0.127 | 0.191 | 0.184 | 0.098 | 0.246 | 0.272 | 0.256 |
| *Ruminococcus gnavus* group | 0.073 | 0.682 | 0.158 | 0.497 | 0.138 | 0.253 | 0.123 | 0.134 | 0.097 | 0.067 | 0.068 | 0.098 | 0.092 |
| *Ruminococcus torques* group | 2.603 | 1.245 | 1.986 | 0.993 | 1.397 | 1.014 | 1.006 | 5.741 | 3.286 | 1.807 | 3.173 | 2.503 | 4.402 |
| *Subdoligranulum* | 3.216 | 0.303 | 0.201 | 0.275 | 0.198 | 0.251 | 0.187 | 0.282 | 0.245 | 0.206 | 0.335 | 0.337 | 0.281 |
| *Lactobacillus* | 0.009 | 0.015 | 0.013 | 0.008 | 0.003 | 0.008 | 0.002 | 0.013 | 0.018 | 0.011 | 0.003 | 0.005 | 0.006 |

**Figure S1**. PCA scatter plot of genera with abundance higher than 0.1% in the inoculum (InInf) and samples from R1, R2, R3 at the end of the first (S1) and second week (S2) of stabilization in the BFBL gut model, at the end of the probiotic supplementation (PR1, PR2, PR3), and at the end of the wash-out (w/o) period.

Fig. S1. Scatterplot microbiota


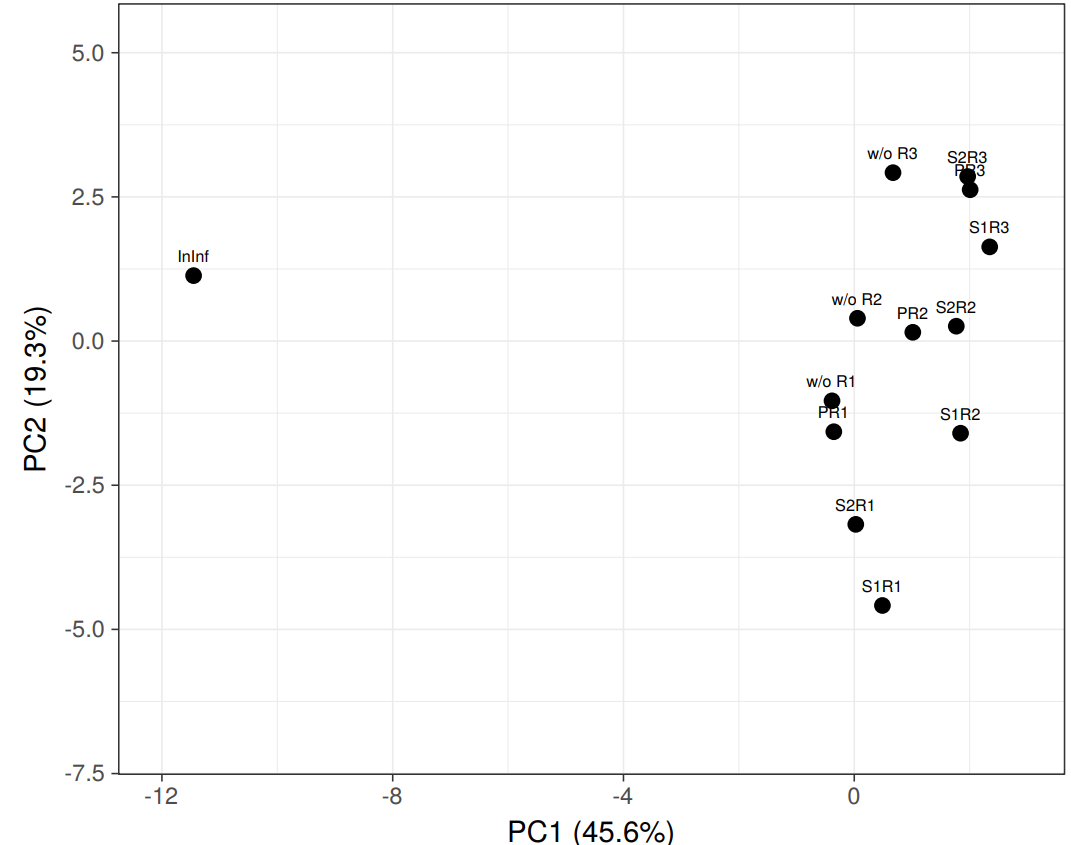

Supplement: Supplementary file 1 — Supplementary file1 (DOCX 215 kb) [file 12602_2024_10280_MOESM1_ESM.docx]
